# Mixed conifer-broadleaf trees on arbuscular mycorrhizal and ectomycorrhizal communities in rhizosphere soil of different plantation stands in the temperate zone, Northeast China

**DOI:** 10.3389/fmicb.2022.986515

**Published:** 2022-09-27

**Authors:** Yong Zhang, Qingcheng Wang, Liqing Xu, Shuangjiao Ma, Donghai Cui, Kaiyue Zhu, Wanju Feng

**Affiliations:** Key Laboratory of Sustainable Forest Ecosystem Management-Ministry of Education, School of Forestry, Northeast Forestry University, Harbin, China

**Keywords:** arbuscular mycorrhizal fungi, ectomycorrhizal fungi, mixed plantation, colonization rate, diversity, community dissimilarity

## Abstract

In comparison with ectomycorrhizal (EM) tree species, arbuscular mycorrhizal (AM) trees have different litter quality and nitrogen cycle modes, which may affect mycorrhizal colonization and the community composition and diversity. However, available studies addressing the mycorrhizal fungal colonization rate, diversity and community composition in mixed forest stands composed of AM and EM trees are rare. In the present study, we assessed litter quality, soil physicochemical properties and correlated them with mycorrhizal community characteristics in rhizosphere soils of monoculture and mixture plantation stands of AM tree species (*Fraxinus mandschurica* Rupr.) and EM tree species (*Larix gmelinii* Rupr., *Picea koraiensis* Nakai) in Northeast China. We hypothesized that (1) the effect of mixture pattern on mycorrhizal colonization rate and diversity would change with tree species, (2) the effect of mixture pattern on mycorrhizal community composition would be less pronounced in comparison with that of tree species. We found that mixture did not change AMF colonization rate regardless of mixture identity, whereas mixture and tree species exerted significant effects on EMF colonization rate. For AMF community, both M-AS (*Fraxinus mandschurica* Rupr. and *Picea koraiensis Nakai*) and M-AL (*Fraxinus mandschurica* Rupr. and *Larix gmelinii* Rupr.) mixtures significantly increased Pielou index and Simpson index, whereas only M-AS significantly increased Sobs. For EMF community, mixture significantly affected examined diversity indices except for Chao1. Mixture significantly shifted AMF and EMF community, and the magnitude was tree species dependent. The dominant genera in AMF and EMF communities in plantation stands were *Glomus* and *Tomentella*, respectively. The EnvFit analysis showed that the determinant factors of EMF community are soil moisture, pH, nitrate nitrogen content, dissolved organic nitrogen content, soil organic matter content, soil organic carbon/total nitrogen and litter carbon/total nitrogen. In conclusion, mixed conifer-broadleaf trees significantly changed soil physicochemical properties, litter quality as well as mycorrhizal fungi community diversity and composition.

## Introduction

A mycorrhizal association is a symbiotic relationship that occurs between plant roots and soil fungi that increases plant performance by enabling plants to extract nitrogen (N) and phosphorus (P) more efficiently from the soil, improving plant productivity and resistance to pathogens, which is extensively distributed in forest ecosystems (Van der Heijden et al., 2015). Arbuscular mycorrhizal fungi (AMF) or ectomycorrhizal fungi (EMF) form a symbiotic relationship with almost every tree species (Brundrett and Tedersoo, 2018; Averill et al., 2022). As a vital component of N cycling in ecosystems, AMF and EMF are responsible for 20 and 80% of plant N uptake, respectively, (Leigh et al., 2009; Van der Heijden et al., 2015). However, AMF and EMF have completely distinct strategies for utilizing N. For example, the AMF community, primarily relies on inorganic N as a source of N in soil (Phillips et al., 2013), whereas the EMF can utilize organic N directly from decomposing organic matter (Read and Perez-Moreno, 2003; Van der Heijden et al., 2015). In addition, AMF and EMF communities respond differently to soil N availability (Phillips et al., 2013; Aldrich-Wolfe et al., 2020).

According to the mycorrhizal type, the trees can be classified into arbuscular mycorrhizal (AM) and ectomycorrhizal (EM) trees. Different mycorrhizal tree species produce different litter qualities that affect soil N content (Phillips et al., 2013). AM tree species generally have a quality litter of a lower carbon to nitrogen ratio (C/N) than EM ones (Phillips et al., 2013; Albornoz et al., 2016; Taylor et al., 2016; Lin et al., 2017; Seyfried et al., 2021; Prada et al., 2022). As proposed by the mycorrhizal-associated nutrient economy theory, forests of AM trees dominate by an inorganic N cycle (Phillips et al., 2013), characterizing by higher soil inorganic N concentration and higher N cycling rate (Lin et al., 2017). Numerous studies found that content of N not only affects colonization rate (Johnson et al., 2003; ÖPik et al., 2010; Sun et al., 2010; Witte et al., 2017; Frater et al., 2018; Lu et al., 2020; Yang et al., 2021a), but also richness, diversity and community composition of AMF (Roy et al., 2017; Witte et al., 2017; Aldrich-Wolfe et al., 2020; Luo et al., 2020; Yang et al., 2021a; Boeraeve et al., 2022). In general, enhanced N availability induce a decline in mycorrhizal association, because of a reduction in plant carbon allocation to root and soil microorganisms (Högberg et al., 2021; Ma et al., 2021).

The plantations acreage in China is the largest in the world. Because of monoculture and high density planting, most plantations are encountering ecological problems, such as soil degradation, productivity reductions, and biodiversity loss (Wang et al., 2013; Felton et al., 2016; Pretzsch et al., 2017; Yang et al., 2017; Wu et al., 2019). In contrast, mixed plantations increase soil microbial diversity and enzyme activity, facilitate litter decomposition, soil fertility improvement and forest productivity (Seidel et al., 2013; Forrester, 2014; Lang et al., 2014; Wu et al., 2019). Earlier studies found that the spore density, community diversity, and abundance of AM fungi in mixed forests were significantly higher than those in monoculture forests (ÖPik et al., 2010; Li et al., 2021a). In contrast, mixed *Juglans mandshurica* and *Larix gmelinii* did not change AM fungal richness and diversity, but significantly affected AM fungal community structure and composition (Ji et al., 2021). To date, most of the available studies focus on assessing the effects of tree monoculture on mycorrhizal fungal colonization and communities associated with a single mycorrhiza type (Aldrich-Wolfe et al., 2020; Yang et al., 2021a; Wang and Han, 2022). In contrast to the growing interest in comparing AM and EM in terms of their functions and responses to environmental factors (Kilpeläinen et al., 2019), there is still a great knowledge gap about the potential effects of tree mixture comprising of AM and EM tree species on mycorrhizal fungal community.

The objective of this study was to determine whether and how soil nutrient differences and litter quality due to mixing AM with EM tree species would affect mycorrhizal fungi communities in 33-year-old mixture and monoculture plantation forest stands of *Fraxinus mandschurica* Rupr., *Larix gmelinii* Rupr., and *Picea koraiensis* Nakai (Jiang et al., 2020; Zarif et al., 2020). We hypothesized that (1) the effect of mixture pattern on mycorrhizal colonization rate and diversity would change with tree species, (2) the effect of mixture pattern on mycorrhizal community composition would be less pronounced in comparison with that of tree species. We further assumed that colonization rates, richness and diversity of AMF communities in the mixture would increase whereas the colonization rate, richness and diversity of EMF in the mixture would decline because of litter quality and soil N availability. Meanwhile, we predicted that AM and EM fungal community composition in the mixture would shift due to changes in soil physicochemical properties and litter quality.

## Materials and methods

### Site description

A study was conducted at the Maoershan Experimental Station of Northeast Forestry University, Heilongjiang province, China (45°25′24′′N, 127°38′56′′E, 400–500 m elevation; Supplementary Figure S1), which is located at the branch range of the Changbai Mountains, with a landform feature of low mountains and hills. This region has a temperate continental monsoon climate with annual average temperatures of 2.8°C and monthly average temperatures of −19.6°C and 20.9°C. The annual average precipitation is approximately 600–800 mm, and there are 120–140 days without frost each year (Zarif et al., 2020). Soils at the site are Hap-Boric Luvisol, which includes loamy soil with a depth of 1–10 cm and sandy loam with a depth of 10–20 cm (Yang et al., 2015). This forest is considered to be a secondary forest. The dominant species are *Betula platyphylla*, *Fraxinus mandshurica*, *Picea koraiensis*, *Populus davidiana*, *Pinus sylvestris* var. *mongolica*, *Larix gmelini*, *Juglans mandshurica* and *Quercus mongolica* (Yang et al., 2010).

### Field sampling

Sampling was conducted in five plantation stands, including Ash (*Fraxinus mandschurica* Rupr.) monoculture (P-A), Larch (*Larix gmelinii* Rupr.) monoculture (P-L), Spruce (*Picea koraiensis* Nakai) monoculture (P-S), Ash and Larch mixture (M-AL), Ash and spruce mixture (M-AS). These stands were first established in 1987 by planting on a strip cutover site of a secondary forest with similar site conditions. The initial spacing between two adjacent trees was 1.5 m × 2.0 m. In the mixtures, broad-leafed trees planted in 3 rows and coniferous ones planted in 5 rows alternatively. There are three replications for each stand. The current features of the stands are given in Table 1. In July 2020, the plots were set up according to the method as described by Luo et al. (2020), with appropriate adjustments. Each replication was considered a 30 m × 50 m plot, 50 m apart from one another. In each plot, nine medium-sized trees, each 10 m apart, were randomly selected from a monoculture, and nine trees adjacent to another tree species were randomly selected for each species in the mixture. Three litter collecting nets were set up 5 m away from selected trees in each plot, and litter was collected in October 2020.

**Table 1:** The features of AM, EM tree species monoculture and mixture plantation stands in Maoershan Experiment Station, Northeast China.

Stand	Species composition	Mycorrhizal type	Height (m)	DBH (cm)	Canopy closure	Litter thickness (cm)	Humus depth (cm)
P-A	Ash	AM	15.86	13.70	0.9	2.1	7.5
M-AL	Ash+Larch	AM-EM	Ash:19.89	Ash:14.75	0.8	3.3	6.3
			Larch:21.18	Larch:18.67			
M-AS	Ash+Spruce	AM-EM	Ash:13.93	Ash:12.71	0.9	3.9	6.9
			Spruce:13.12	Spruce:15.35			
P-L	Larch	EM	18.91	20.51	0.8	3.6	6.5
P-S	Spruce	EM	13.31	16.05	0.9	5.1	5.9

DBH, diameter at breast height; P-A, Ash monoculture; M-AL, Ash-Larch mixture; M-AS, Ash-Spruce mixture; P-L, Larch monoculture; P-S, Spruce monoculture.

Root and soil samples were collected from the midpoint between selected trees and the neighbors in monocultures and between selected trees and the neighboring trees of another species in mixtures. First-order root samplings and rhizosphere soil samplings were taken using the methods described previously (Phillips and Fahey, 2006; Chen et al., 2018). Before sampling, litter on the soil surface around the trees was removed and then the first-order roots were carefully traced along the main root of the tree by a hand shovel. At least 100 first-order roots were taken from each sample tree. After gently shaking the fine roots, the soil loosely and tightly attached to the root system was taken by handshaking and brushing, respectively, and was defined as rhizosphere soil. Rhizosphere soil samples were taken from each sample tree within a 0–10 cm soil depth. Bulk soil, i.e., soil not adhering to root, was also sampled within the same soil depth. Nine root samples, nine rhizosphere soil samples and nine bulk soil samples of each tree species in each plot (3 replicated plots) were pooled to produce plot a level sample, respectively (Han et al., 2020; Luo et al., 2020). The root and soil samples were cold stored in a cooler and taken to the lab in no time.

Root samples were rinsed by deionized water, the living roots were selected and fixed in formalin-aceto-alcohol (FAA, 90 ml of 70% alcohol, 5 ml of 38% formaldehyde and 5 ml of glacial acetic acid) solution for subsequent determination of mycorrhizal colonization rate (Yang et al., 2021b). Soil samples were sieved with a 2-mm mesh to remove roots and litter. Rhizosphere soil samples were put in a −80°C refrigerator for later DNA detection, and fresh bulk soil of each sample was divided into two parts, one was stored at −20°C in a refrigerator for nitrate N (NO_3_^−^-N), ammonium N (NH_4_^+^-N), and dissolved organic N (DON) assaying, and the other was air-dried and ground for the other variables’ determination.

### Soil chemical analyses

Soil moisture content (SM) was measured using the drying and weighing method. In brief, SM was measured by weighting before and after drying approximate 25 g fresh soils at 105°C overnight. Soil pH was measured in a soil-water suspension at a soil:water ratio of 1:2.5 (Zarif et al., 2020). The contents of nitrate N (NO_3_^−^-N) and ammonium N (NH_4_^+^-N) were extracted by 1 *M* KCl extraction and measured using an AA3 continuous flow analyzer (SEAL AA3, Norderstedt, Germany; Zarif et al., 2020). Litter C/N ratio and soil total N (TN) content were determined by an Elemental Analyzer (Elementar Analysensysteme GmbH, Hanau, Germany). Available P (AP) was extracted by 0.5 *M* NaHCO_3_ and determined using the molybdenum blue method (Watanabe and Olsen, 1965). The contents of dissolved organic N (DON) and organic material (OM) were determined using the methods described by Lu (1999).

### Mycorrhizal colonization rate

First-order root tips of AM and EM tree species were randomly selected from the FAA solution in each plot and stained using the previous method (Phillips, 1970). In brief, root tips were softened using 5% KOH and then heated in water at a constant temperature of 65°C for 5 min. The root tips were cleaned with 1% HCl for 10 min and then stained with 0.05% trypan blue. The AMF colonization rate was estimated according to the gridline intercept method as described by Giovannetti and Mosse (1980). The roots of EMF tree species were observed under the microscope to calculate the EMF mycorrhizal colonization rate, which was calculated by the number of colonized root tips/total number of root tips (Cline et al., 2005; Suz et al., 2014; Nagati et al., 2019).

### DNA extraction and PCR amplification

All genomic DNA samples were distilled using OMEGA Soil DNA Kit (M5635-02; Omega Bio-Tek, Norcross, GA, United States) following the manufacturer’s instructions, and placed at –20°C for the next step of assay. The number and quality of DNA obtained in the previous step were measured using a NanoDrop NC2000 spectrophotometer (Thermo Fisher Scientific, Waltham, MA, United States) and agarose gel electrophoresis, respectively.

Nested PCR was conducted to amplify the AMF gene fragments with high specific amplification. In the first round of amplification, the AML1F (5′-ATCAACTTTCGATGGTAGGATAGA-3′) and AML2R (5′-GAACCCAAACACTTTGGTTTCCTTGGTTTCC-3′) were used as the primers, whereas in the second amplification step, the AMV4.5NF (5′-AAGCTCGTAGTTGAATTTCG-3′) and AMDGR (5′-CCCAACTATCCCTATTAATCAT-3′) (Luo et al., 2020) were used as the primers. For the amplification of EMF, the highly variable ITS1 region of the fungal ITS gene was selected as the target fragment, and fungal ITS5F (5′-GGAAGTAAAAGTCAACAAGG-3′) and ITS1R (5′-GCTGCGTTCTTCATCGATGC-3′) were selected as specific primers (Ma et al., 2022).

PCR amplification: firstly, denature the mold at 98°C for 5 min, followed by denaturation at 98°C for 30 s, then 53°C for 30 s, 72°C for 45 s, allowing the primers to extend on the template and synthesize DNA. The cycle was maintained for 25 times, so that a large amount of amplified DNA fragments accumulated. Final extension was at 72°C for 5 min to make the primer extension complete (Zong et al., 2022). Vazyme V AHTSTM DNA Clean Beads (Vazyme, Nanjing, China) and the Quant-iT PicoGreen dsDNA Assay Kit (Invitrogen, Carlsbad, CA, United States) were used to purify and quantify PCR amplicons. When individual quantification step finished, amplicons were pooled in equal amounts, and pair-end 2 × 250 bp sequencing was performed using the Illumina MiSeq platform with MiSeq Reagent Kit v3 at Shanghai Personal Biotechnology Co., Ltd. (Shanghai, China; Zong et al., 2022).

### Sequence analysis

Following the official course, with minor modifications, microbiome bioinformatics was carried out by QIIME2 2019.4^1^ (Bolyen et al., 2019). In short, the original sequence data were dissected repeatedly using the demux plugin, and then the primers were cut using the cut adapt plugin (Martin, 2011). Then, Sequences were merged, quality filtered and de-replicated with *fastq_mergepairs*, *fastq_filter* and *derep_fullength* in Vsearch plugen. All the unique sequences were clustered at 98% (*via cluster_siz*e), and then chimera was removed (*via uchime_denovo*). Finally, the *non_chimera* sequences were clustered at 97% to generate OTU representative sequences and OUT Table. Non-singleton amplicon sequence variants (OTUs) were aligned using mafft (Katoh, 2002). After the completion of sequence clustering, RDP classifier algorithm was used to classify the taxonomy of each AMF and EMF OTU from the domain level to the OTU level by comparing with MaarjAM database (Maarjam 081; ÖPik et al., 2010) and Unite v8 database (Nilsson et al., 2019), respectively, with the default confidence threshold of 0.7, and OTUs of EMF were distinguished against the FUNGuild database (Nguyen et al., 2016). Sequence data analyses were mainly performed using QIIME2 and R (v3.2.0) packages. Dilution curves were generated to compare the effects of sequencing depth on the diversity of observation samples (QIIME2). The OTU-level diversity, including number of OTUs observed (Sobs), Chao1, Shannon diversity index, Pielou index and Simpson index, were calculated using the OUT table in QIIME2.

### Statistical analyses

The differences in AMF community characteristics, soil chemical properties and litter quality associated with AMF trees across treatments were examined with one-way analysis of variance (one-way ANOVA) or Kruskal-Wallis test. The normality and variance homogeneity were examined by using the Shapiro–Wilk normality test and Levene’s test, respectively. When the assumption met, one-way ANOVA followed by Turkey’s HSD for pair-wise comparisons was used. Otherwise, the Kruskal-Wallis test was used. The differences in EMF community characteristics soil chemical properties and litter quality associated with EMF trees across treatments were examined with Two-way analysis of variance (two-way ANOVA) or Scheirer-Ray-Hare test. Spearman correlations between colonization rates and environmental factors as well as those between alpha (α) diversity indices and environmental factors were investigated using the “pheatmap” package. Venn diagrams were generated to compare the shared and unique OTUs between different stands with the R package, based on the occurrence of OTUs in a sample or group regardless of their relative abundance using VennDiagram. Beta (β) diversity were measured by calculated the Bray-Curtis dissimilarities. In addition, the Bray–Curtis dissimilarity metrics were fit on the nonmetric multidimensional scaling (NMDS) graph using the “vegan” package to show the differences across treatments. Statistical significance was determined by permutational multivariate analysis of variance (PERMANOVA) on Bray–Curtis dissimilarities with 999 permutations to study the effect of different factors on the structure of fungal communities using the “vegan” package. Finally, the major environmental factors shaping soil mycorrhizal community were selected with ‘EnvFit’ function in the ‘vegan’ package of R. All statistical analysis was performed in R (version 4.2.0).

## Results

### Soil physicochemical properties and litter quality

In AM tree stands, soil nitrate nitrogen content, DON, OM, soil C/N and litter C/N were significant changed by mixture identity (Table 2). Specifically, the M-AS significantly reduced soil nitrate nitrogen content, while significantly increased OM content, soil C/N and litter C/N in comparison with the P-A monoculture. DON content was significantly reduced in mixture stands, and its reduction in the M-AS stands was more pronounced than that in the M-AL stands. AP in the M-AS stands was significantly higher than that in the M-AL stands. In EM tree stands, soil pH and OM content were significantly affected by planting pattern, tree species and their interaction, soil nitrate nitrogen content, DON content, soil C/N and litter C/N were significantly affected by planting pattern and tree species, AP was significantly affected by the interaction of planting pattern and tree species (Supplementary Table S1).

**Table 2:** Soil physicochemical properties and litter quality of ash monoculture, Ash-Larch mixture and Ash-Spruce mixture stands at Maoershan Experiment Station, Northeast China.

Variables	P-A	M-AL	M-AS	*df*	*F*	*p*
Soil moisture (%)	36.06 ± 0.35	35.94 ± 0.34	36.47 ± 0.47	2	0.495	0.630
pH	5.86 ± 0.02	5.80 ± 0.02	5.83 ± 0.02	2	1.369	0.320
TN (g/kg)	8.81 ± 1.51	9.40 ± 1.30	9.05 ± 0.90	2	0.165	0.852
NO_3_^−^-N (mg/kg)	29.79 ± 3.58 a	23.33 ± 2.31 a	16.71 ± 1.62 b	2	18.550	0.003
NH_4_^+^-N (mg/kg)	14.00 ± 1.92	10.39 ± 1.49	9.65 ± 1.93	2	5.058	0.520
DON (mg/kg)	18.27 ± 0.41 a	14.39 ± 0.93 b	11.11 ± 0.42 c	2	63.090	< 0.001
OM (g/kg)	97.87 ± 6.31 b	99.34 ± 8.05 b	139.07 ± 3.96 a	2	40.840	< 0.001
AP (mg/kg)	18.83 ± 0.89 ab	17.26 ± 2.75 b	25.21 ± 2.43 a	2	11.440	0.009
Soil C/N	6.52 ± 0.40 b	6.17 ± 0.31 b	8.95 ± 0.38 a	2	17.340	0.003
Litter C/N	21.71 ± 0.63 b	27.88 ± 2.89 b	43.47 ± 1.51 a	2	34.270	< 0.001

All values represent mean ± standard error (SE), with three replicates per treatment. Soil moisture was calculated as per cent dry weight. Different lowercase letters indicate significant differences between different stands by Tukey’s honestly significant difference test (*α* = 0.05). AP, available phosphorus; DON, dissolved organic nitrogen; litter C/N, litter carbon/total nitrogen; NH_4_^+^ -N, ammonium nitrogen; NO_3_^- ^-N, nitrate nitrogen; OM, organic matter; Soil C/N, soil organic carbon/total nitrogen; TN, total nitrogen; P-A, Ash monoculture; M-AL, Ash-Larch mixture; M-AS, Ash-Spruce mixture.

### Colonization rate of mycorrhizal fungi

**Figure 1:**
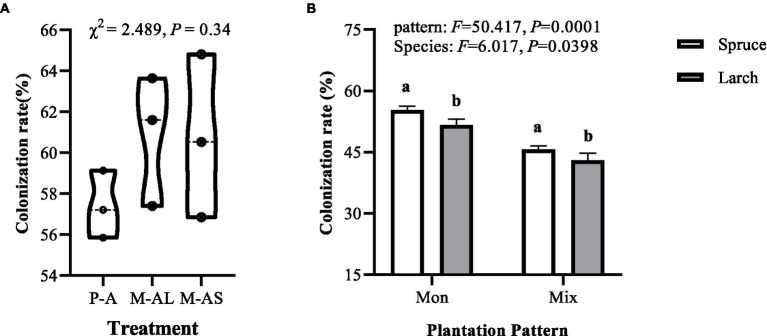
Colonization rate of arbuscular mycorrhizal fungi **(A)** and ectomycorrhizal fungi **(B)** in stands of monocultures and mixtures at the Maoershan Experiment Station, Northeast China. M-AL, Ash-Larch mixture; M-AS, Ash-Spruce mixture; P-L, Larch monoculture; P-S, Spruce monoculture. Mon, monoculture; Mix, mixture. All values represent mean ± standard error (SE), with three replicates per treatment. Different lowercase letters indicate significant differences between different stands according to Tukey’s honestly significant difference test (*α* = 0.05).

AMF colonization rate in roots of forest plantation stands ranging from 55.86 to 64.81%, and no significant difference in the AMF colonization rate across examined stands (Figure 1A, Kruskal-Wallis test, *χ*^2^ = 2.489, *df* = 2, *p* = 0.34). The EMF colonization rate of forest plantation stands ranging from 40 to 57%. Both mixture (*F* = 50.417, *df* = 1, *p* = 0.0001) and tree species (*F* = 6.017, *df* = 1, *p* = 0.0398) exerted significant effects of colonization rate of EMF community. Specifically, the colonization rate of EMF community in mixture was significantly lower than that of monoculture for the same tree species, and the colonization rate of EMF community in spruce stand was significantly higher than that of larch stand under identical plantation pattern. Further analysis showed that EMF colonization rate was significantly positively correlated to OM content and litter C/N, whereas negatively correlated to soil pH, nitrate nitrogen content and DON content (Table 3; Supplementary Table S1).

### Diversity of mycorrhizal fungi

**Figure 2:**
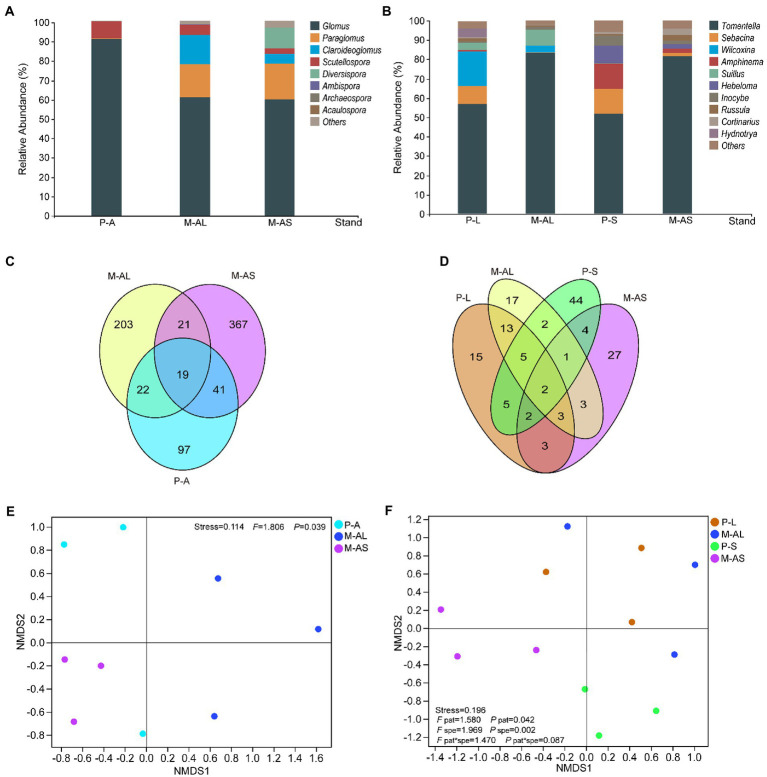
The relative abundance of genera of arbuscular mycorrhizal fungi **(A)** and ectomycorrhizal fungi **(B)** communities, venn diagram displaying the shared OTUs in arbuscular mycorrhizal fungi **(C)** and ectomycorrhizal fungi **(D)** communities as well as NMDS analysis of arbuscular mycorrhizal fungi **(E)**, ectomycorrhizal fungi **(F)** communities in rhizosphere soils of plantation stands at the Maoershan Experiment Station, Northeast China. P-A, Ash monoculture; M-AL, Ash-Larch mixture; M-AS, Ash-Spruce mixture; P-L, Larch monoculture; P-S, Spruce monoculture; pat, pattern; spe, species.

For AMF community, both M-AS and M-AL mixture significantly increased Pielou index and Simpson index, whereas only M-AS significantly increased Sobs (Table 4). All diversity indices significantly positively correlated to litter C/N, while significantly negatively correlated to DON and soil nitrate nitrogen content (Supplementary Figure S2A). For EMF community, mixture significantly affected examined diversity indices except for Chao1, regardless of tree species (Figure 2; Supplementary Table S2). The Sobs significantly positively correlated to litter C/N, while significantly negatively correlated to soil pH. The Pielou index significantly positively correlated to litter C/N. The Shannon index significantly positively correlated to litter C/N and soil OM content, whereas significantly negatively correlated to soil pH and soil nitrate nitrogen content. The Simpson index significantly positively correlated to litter C/N and soil C/N, whereas significantly negatively correlated to soil nitrate nitrogen content, DON content and pH (Supplementary Figure S2B).

### Community structure and composition

By clustering OTU at a 97% similarity threshold, we identified 770 AMF OTUs and 146 EMF OTUs. The representative AMF and EMF OTUs were associated with 1 phylum, 3 classes, 4 orders, 8 families, 8 genera and 2 phyla, 4 classes, 11 orders, 12 families, 12 genera, respectively. As indicated by the gentle dilution curve, the sequencing depth was the sequencing depth was adequate for evaluating the structure and diversity of mycorrhizal fungi across all samples (Supplementary Figure S3). For AMF community in the rhizosphere soils, the dominant genus of all plantation stands was the *Glomus* (Figure 2A). Its relative abundances were 90.89 ± 8.58 (%), 60.89 ± 19.2 (%) and 59.84 ± 0.86 (%), respectively. In addition, the relative abundance of the *Paraglomus* was relatively high in some rhizosphere soil samples of the M-AL and M-AS mixture stands. For EMF community, the dominant genera and their relative abundances varied with plantation pattern and tree species. In rhizosphere soils of all examined plantation stands, the *Tomentella* was the dominant genus (Figure 2B). Its relative abundances in rhizosphere soils of the M-AL and M-AS stands were 83.41 ± 8.78 (%) and 81.65 ± 4.63 (%), respectively (Figure 2B). In contrast, the relative abundances of the *Tomentella* genus in rhizosphere soils of P-L and P-S were 57.19 ± 4.62 (%) and 51.72 ± 13.29 (%), respectively. As shown in the Veen plots, 19 AMF OTUs were shared by the monoculture and mixture stands, and the number of unique OTU in mono stands was also much lower than that in the mixed stands (Figure 2C). A similar result was observed for EMF. Specifically, the number of EMF OTUs shared by M-AL and P-L, M-AS and P-S stands were 23 and 9, respectively, while there were only 2 shared EMF OTUs in four stands (Figure 2D). The NMDS displayed that AMF community composition in the rhizosphere soil under AM monoculture and AM-EM mixtures differed significantly (Figure 2E), the same is true for EMF community composition (Figure 2F). Further analysis showed that the determinant factors of EMF community are soil moisture, pH, nitrate nitrogen content, DON content, OM content, soil C/N and litter C/N (Supplementary Table S3).

## Discussion

Since AM and EM tree species usually coexist (Kubisch et al., 2016), this study investigated the effects of mixed conifer-broadleaf trees on mycorrhizal community in rhizosphere soils of AM and EM tree species, which differ in litter quality and N utilizing strategy in Northeast, China by the Illumina MiSeq sequencing. Our results showed that mixed conifer-broadleaf differentially affected the soil physicochemical properties and the diversity, community structure and composition of AMF and EMF.

### Litter quality and soil physicochemical properties

As hypothesized, mixture of conifer-broadleaf trees changed soil physicochemical properties and litter quality (Tables 2, 3). Previous studies suggested that difference in plant species composition and litter diversity contributes to changes in patterns and rates of litter decomposition by microbial community in forests (ÖPik et al., 2010). In general, litter C/N ratio of EM trees is significantly higher than that of AM trees (Phillips et al., 2013), and thus difficult for microorganisms to utilize and decompose (Zeng et al., 2014), resulting in a low contribution to soil N availability content (Phillips et al., 2013; Lin et al., 2017; Witte et al., 2017). Moreover, Cremer and Prietzel (2017) found that mixed conifer-broadleaf tree litter had a significant effect on soil pH, which depends on organic acid production resulting from litter decomposition. In agreement with these findings, the litter C/N ratio of the mixtures was higher than that of EM monocultures and lower than that of AM monoculture, whereas the opposite held true for soil N availability in the present study (Tables 2, 3). In combination with Li et al. (2021b), we observed mixture significantly reduced pH but only in spruce monoculture stands (Table 3), indicating the effect of mixture on soil pH depends on mixture identity and tree species.

**Table 3:** Soil physicochemical properties and litter quality of Larch monoculture, Spruce monoculture, Ash-Larch mixture and Ash-Spruce mixture stands at Maoershan Experiment Station, Northeast China.

Variables	M-AL	M-AS	P-L	P-S
Soil moisture (%)	35.94 ± 0.34 Aa	36.47 ± 0.47Aa	34.98 ± 0.36 Aa	35.57 ± 0.77Aa
pH	5.80 ± 0.02 Aa	5.83 ± 0.02 Aa	5.75 ± 0.03 Aa	5.60 ± 0.04 Bb
TN (g/kg)	9.40 ± 1.30 Aa	9.05 ± 0.90 Aa	8.34 ± 1.06 Aa	8.71 ± 1.79 Aa
NO_3_^−^-N (mg/kg)	23.33 ± 2.31 Aa	16.71 ± 1.62 Ab	17.73 ± 1.94 Ba	13.04 ± 1.40 Aa
NH_4_^+^-N (mg/kg)	10.39 ± 1.49 Aa	9.65 ± 1.93 Aa	9.96 ± 0.85 Aa	6.90 ± 1.28 Aa
DON (mg/kg)	14.39 ± 0.93 Aa	11.11 ± 0.42Ab	12.21 ± 0.66 Ba	9.35 ± 0.83 Ab
OM (g/kg)	99.34 ± 8.05 Ab	139.07 ± 3.96 Ba	111.05 ± 8.33 Ab	173.52 ± 3.22 Aa
AP (mg/kg)	17.26 ± 2.75 Aa	25.21 ± 2.43 Aa	23.17 ± 1.01 Aa	19.83 ± 3.00 Aa
Soil C/N	6.17 ± 0.31 Aa	8.95 ± 0.38 Ba	7.79 ± 0.52 Aa	11.90 ± 2.50 Aa
Litter C/N	27.88 ± 2.89 Bb	43.47 ± 1.51 Bb	46.98 ± 1.57 Aa	64.23 ± 2.14 Aa

All values represent mean ± standard error (SE), with three replicates per treatment. Soil moisture was calculated as per cent dry weight. Different uppercase letters indicate significant differences between patterns at *α* = 0.05 level, different lowercase letters indicate significant differences between tree species at *α* = 0.05 level. AP, available phosphorus; DON, dissolved organic nitrogen; Litter C/N, litter carbon/total nitrogen; NH_4_^+^ -N, ammonium nitrogen; NO_3_^- ^-N, nitrate nitrogen, OM, organic matter; Soil C/N, soil organic carbon/total nitrogen; TN, total nitrogen; M-AL, Ash-Larch mixture; M-AS, Ash-Spruce mixture; P-L, Larch monoculture; P-S, Spruce monoculture.

### Colonization rate of mycorrhizal fungi

Previous studies suggested that differences in soil N content will lead to significant changes in mycorrhizal colonization (Blanke et al., 2005), and increased soil N content are expected to reduce AMF colonization rate (e.g., Johnson et al., 2003; Johnson, 2010; Frater et al., 2018) because host plants will allocate less carbon to AMF when belowground resources no longer constrain plants (Johnson, 2010; Chen et al., 2017). Furthermore, AMF abundance was negatively correlated with soil nitrate nitrogen content (Cui et al., 2016). Similarly, enhanced N availability is also detrimental for EMF colonization (ÖPik et al., 2010; Witte et al., 2017; Yang et al., 2021a). In the present study, we found no significant effect of mixture on AMF colonization rate (Figure 1A). Additionally, we found mixed conifer-broadleaf trees significantly changed soil nitrate nitrogen content and DON content (Table 2), whereas no significant association between the AMF colonization rate and environmental factors including litter C/N and soil physicochemical properties were found in our study (Supplementary Figure S2A). This implies there maybe another important factors influencing AMF colonization, further study should be conducted. However, we found EMF colonization was significantly lower in mixtures than in monoculture for the same tree species and negatively correlated with soil nitrate nitrogen content and DON content in this study (Figure 1B; Supplementary Figure S2B). With an increase in soil N availability, host plants obtain enough nitrogen with less carbon input, resulting in a decrease in EMF colonization (Sun et al., 2010).

### Diversity of mycorrhizal fungi

In this study, we found mixture did not affect the Chao1 for AMF and EMF communities, whereas the diversity change magnitude depends on the target index and mixture identity (Table 4; Supplementary Table S2). Our findings are contrast to the reports that addressing the mixed conifer-broadleaf trees on soil fungal diversity and richness (Wu et al., 2019; Ji et al., 2021; Li et al., 2021b). Wu et al. (2019) found that diversity indices were higher in soil samples from mixed plantations than in those from corresponding pure forests. Ji et al. (2021) found that the diversity and richness in soil samples from mixed plantations were comparable to those from corresponding pure forests. Li et al. (2021b) reported that the Chao1 index of EMF was responsive to mixture. The discrepancy in the effects of mixture on mycorrhizal fungal community richness and diversity across studies maybe correlated to tree species, mycorrhizal type, soil nutrient, soil fauna and plant–soil organism interactions. In agreement with previous studies that EMF richness and diversity are negatively correlated to soil nutrient content (Corrales et al., 2016; Erlandson et al., 2016), particularly the N availability (Lilleskov et al., 2011; Hasselquist and Högberg, 2014; Yang et al., 2021a; Boeraeve et al., 2022).

**Table 4:** AMF alpha diversity indices of Ash monoculture, Ash-Larch mixture and Ash-Spruce mixture stands at Maoershan Experiment Station, Northeast China.

Variables	P-A	M-AL	M-AS	*df*	Statistics	*p*
Chao1	107.86 ± 10.03	137.09 ± 15.19	220.16 ± 36.78	2	χ^2^ = 5.689	0.059
Sobs	68.13 ± 9.52 b	103.87 ± 15.76 ab	173.70 ± 35.87 a	2	*F* = 5.320	0.047
Pielou	0.19 ± 0.02 b	0.32 ± 0.03 a	0.31 ± 0.03 a	2	*F* = 5.479	0.044
Shannon	1.19 ± 0.19	2.13 ± 0.26	2.34 ± 0.34	2	*F* = 5.094	0.051
Simpson	0.49 ± 0.05 b	0.69 ± 0.03 a	0.69 ± 0.05 a	2	*F* = 6.885	0.028

All values represent mean ± standard error (SE), with three replicates per treatment. Different lowercase letters indicate significant differences between different stands according to Tukey’s honestly significant difference test (*α* = 0.05). P-A, Ash monoculture; M-AL, Ash-Larch mixture; M-AS, Ash-Spruce mixture.

### Community structure and composition of mycorrhizal fungi

In agreement with our prediction that AM and EM fungal community composition in the mixture would shift due to changes in soil physicochemical properties and litter quality. Previous studies demonstrate that the identities of mixed species are determinant of AMF community in rhizosphere soil (Mummey and Rillig, 2006; Turrini et al., 2016; Ji et al., 2021) and changes in soil properties result in shifts in mycorrhizal fungal communities (Avis et al., 2003; Roy et al., 2017; Chen et al., 2018; Lu et al., 2020; Gu et al., 2022). We found strikingly differences in the AMF communities between M-AL and P-A stands, as well as in the EMF communities between M-AS and P-S stands (Figure 2).

In line with previous studies (Wang et al., 2018; Zeng et al., 2021; Wang and Han, 2022), the *Glomus* and the *Tomentella* were the most abundant genera in AMF and EMF communities, respectively. The *Glomus* was primarily distributed in the upper soil layer, particularly where the available N content was high (Wang et al., 2018; Chen et al., 2021). We found the relative abundance of *Glomus* was higher in AM monoculture, and the relative abundance was negatively correlated to nitrate nitrogen. What accounts for this discrepancy is unknown but warrants further studying. The *Tomentella* has a strong adaptability to the environment and strong colonization ability (Nara, 2006; ÖPik et al., 2010). Meanwhile, more abundance and species of the *Tomentella* was found in soils with high N than that with low N (Kranabetter et al., 2015; Wang and Han, 2022). Besides, EM root tips colonized by *Tomentella* are capable of producing extracellular enzymes used to break down proteins, polysaccharides, and organic forms of P (Courty et al., 2005; Tedersoo et al., 2012) and mobilizing organic N (Pena et al., 2013). We found that mixture enhanced the relative abundance of *Tomentella* (Figure 2B), indicating it would increase the nutrient availability.

Finally, we found that the determinant factors of EMF community are soil moisture, pH, nitrate nitrogen content, dissolved organic nitrogen content, soil organic matter content, soil organic carbon/total nitrogen and litter carbon/total nitrogen. In earlier studies, soil moisture (Erlandson et al., 2016), soil organic carbon (Yang et al., 2019; Li et al., 2021a), nitrate nitrogen (He et al., 2016), soil C/N (Zhang et al., 2013; Sterkenburg et al., 2015), pH (Bahnmann et al., 2018; Craig et al., 2018) and litter C/N (Hewitt et al., 2017) are found to be the critical factors shaping fungal community composition. In addition, soil pH, soil organic matter, total nitrogen, C/N ratio, and total phosphorus influenced the EMF community structure (Li et al., 2021b). In general, our findings again highlight the linkage between soil physicochemical properties and EMF community composition.

The major limitation of the present studies is that we did not consider the seasonal variation. In addition, we only compared the AMF and EMF with a few tree species with limited samples. To draw a general conclusion about responses of mycorrhizal community to conifer-broadleaf mixture, future studies should be conducted under different conditions, such as host plants, climates, soil texture and altitudes. Finally, we did not consider the effects of changes in biological factors, including species and diversity of aboveground plants, root exudates and other soil fauna belowground.

## Conclusion

In the present study, changes in soil physicochemical properties and litter quality due to mixed planting influenced the mycorrhizal colonization rate, composition, and diversity of the rhizosphere mycorrhizal community, especially the EMF community. The determinant factors of EMF community were soil moisture, pH, nitrate nitrogen content, dissolved organic nitrogen content, soil organic matter content, soil organic carbon/total nitrogen and litter carbon/total nitrogen. These results will assist in understanding and predicting variations in mycorrhizae in forest ecosystems composed of different tree species associated with different mycorrhizae. These results provide a better understanding of the ecological functions of the soil–fungal community between mixed and pure plantations, and cast a new light on underlying mechanisms between soil nutrients and soil–fungal functional community, especially for those tree species used in forest plantation.

## Data availability statement

The datasets presented in this study can be found in online repositories. The names of the repository/repositories and accession number(s) can be found at: https://www.ncbi.nlm.nih.gov/genbank/, PRJNA846491 https://www.ncbi.nlm.nih.gov/genbank/, PRJNA846556.

## Author contributions

All authors contributed to the study conception and design. The field and laboratory work were carried out by YZ, LX, SM, DC, and KZ. The first draft of the manuscript was written by YZ and revised by QW and WF. All authors commented on previous versions of the manuscript. All authors contributed to the article and approved the submitted version.

## Funding

This work was supported by the Fundamental Research Funds for the Central Universities of China (2572017PZ03, 2572020DR04, and 2572019CP16).

## Conflict of interest

The authors declare that the research was conducted in the absence of any commercial or financial relationships that could be construed as a potential conflict of interest.

## Publisher’s note

All claims expressed in this article are solely those of the authors and do not necessarily represent those of their affiliated organizations, or those of the publisher, the editors and the reviewers. Any product that may be evaluated in this article, or claim that may be made by its manufacturer, is not guaranteed or endorsed by the publisher.

## Footnotes

^1^https://docs.qiime2.org/2019.4/tutorials/ (accessed September 13, 2022).
