# Supplementary material for: Mixed conifer-broadleaf trees on arbuscular mycorrhizal and ectomycorrhizal communities in rhizosphere soil of different plantation stands in the temperate zone, Northeast China
Source: Front Microbiol. 2022 Sep 27;13:986515. doi: 10.3389/fmicb.2022.986515 (PMC9551461; doi:10.3389/fmicb.2022.986515)
Supplement: Supplementary file 1 [file Data_Sheet_1.docx]

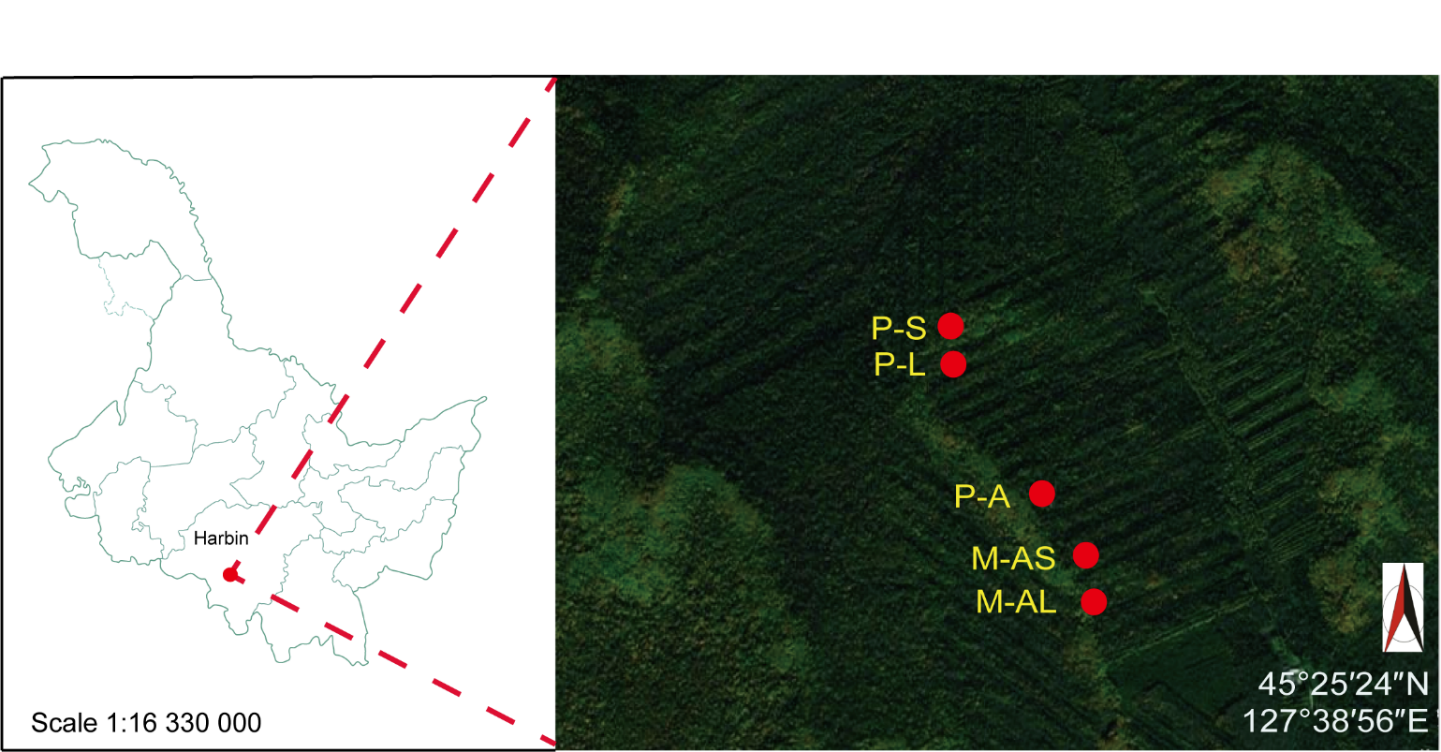


**Supplementary Figure S1** Map of study site in Heilongjiang province, China. The geographic map was generated online at National Platform for Common Geospatial Information Services (https://www.tianditu.gov.cn). P-A, Ash monoculture; M-AL, Ash-Larch mixture; M-AS, Ash-Spruce mixture; P-L, Larch monoculture; P-S, Spruce monoculture.


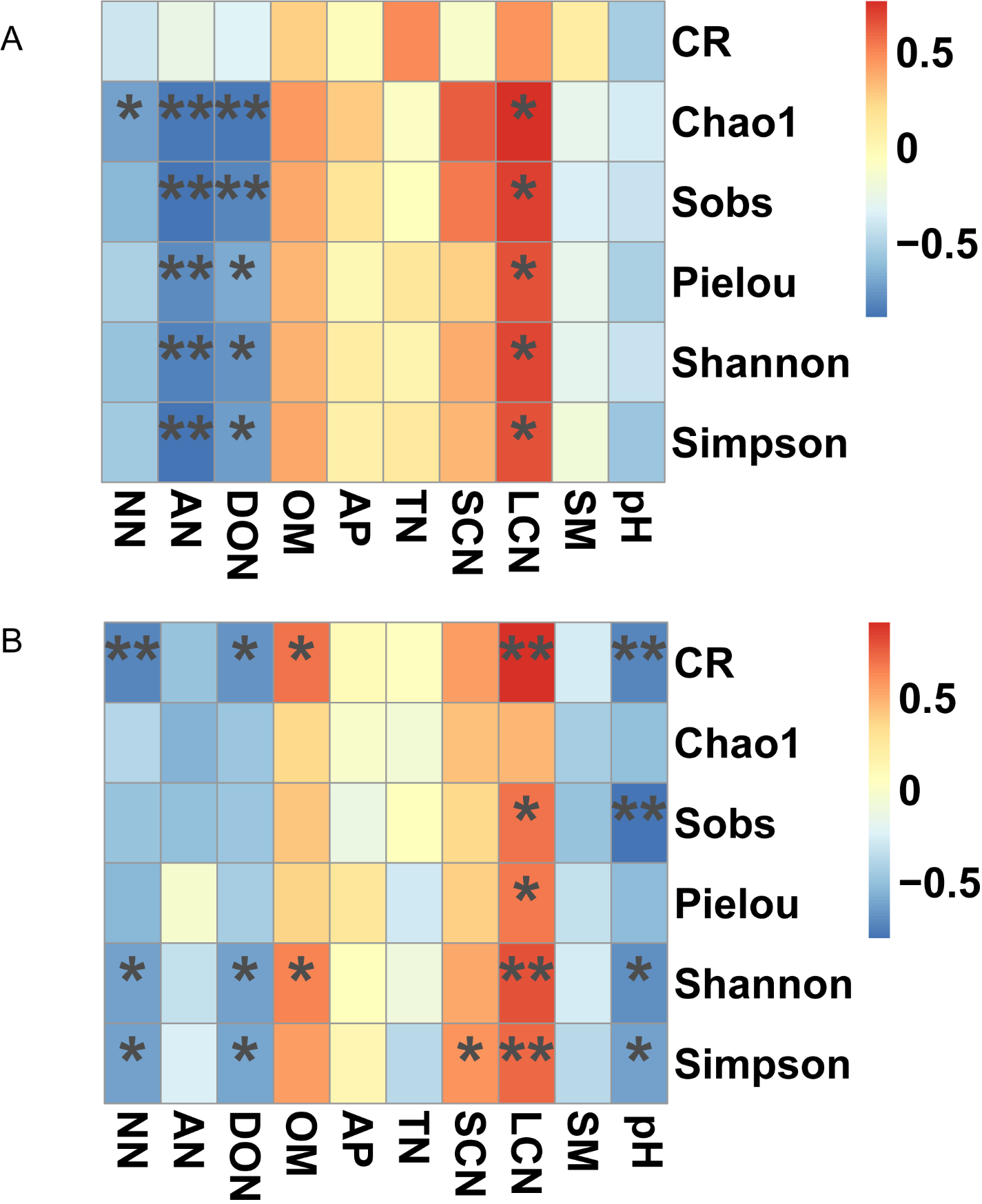


**Supplementary Figure S2** The Spearman correlation analysis between environmental factors (including litter quality and soil physicochemical properties) and arbuscular mycorrhizal fungi (**A**) and ectomycorrhizal fungi (**B**) in rhizosphere soils of plantation stands. **: *p<*0.01; *: *p<*0.05. NN, Nitrate nitrogen; AN, Ammonium nitrogen; DON, Dissolved organic nitrogen; OM, Organic matter; AP, Available phosphorus; TN, Total nitrogen; SCN, Soil organic carbon/total nitrogen; LCN, litter carbon/ nitrogen; SM, Soil moisture; CR, colonization rate.

**
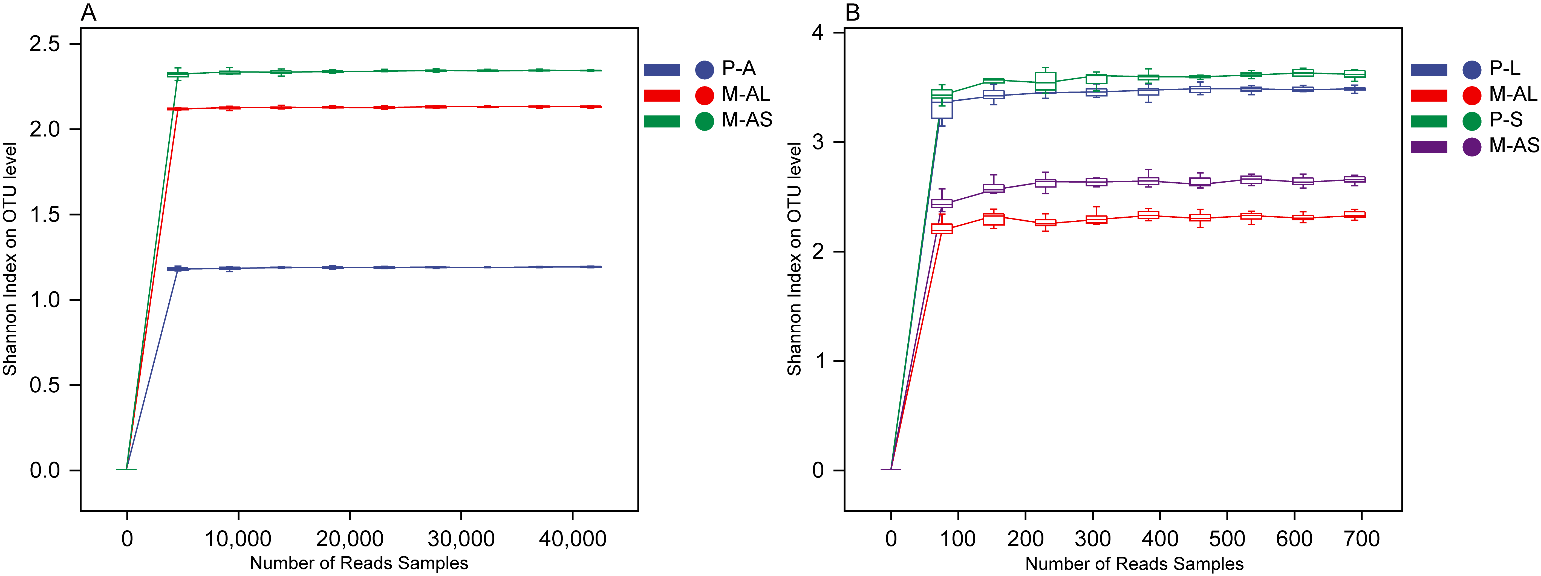
**

**Supplementary Figure S3** Dilution curves of arbuscular mycorrhizal fungi (**A**) and ectomycorrhizal fungi (**B**) in stands of AM, EM monocultures and AM-EM mixtures in Maoershan Experiment Station, Northeast China. P-A, Ash monoculture; M-AL, Ash-Larch mixture; M-AS, Ash-Spruce mixture; P-L, Larch monoculture; P-S, Spruce monoculture.

**Supplementary Table S1** Results of two-way ANOVAs for the effects of pattern and tree species on soil physicochemical properties of Larch monoculture, Spruce monoculture, Ash-Larch mixture and Ash-Spruce mixture stands at Maoershan Experiment Station, Northeast China.

| Factor | Variable | SM | pH | TN | NO_3_^-^-N | NH_4_^+^-N | DON | OM | AP | Soil C/N | Litter C/N |
| --- | --- | --- | --- | --- | --- | --- | --- | --- | --- | --- | --- |
| pattern | *F* | 5.176 | 37.657 | 0.087 | 18.885 | 3.643 | 21.392 | 39.914 | 0.036 | 8.030 | 108.834 |
|  | *P* | 0.053 | <0.001 | 0.378 | 0.002 | 0.092 | 0.002 | <0.001 | 0.855 | 0.022 | <0.001 |
| species | *F* | 1.872 | 6.095 | 0.000 | 23.030 | 5.203 | 51.951 | 195.589 | 2.713 | 18.293 | 73.875 |
|  | *P* | 0.208 | 0.039 | 0.988 | <0.001 | 0.052 | <0.001 | <0.001 | 0.138 | 0.003 | < 0.001 |
| pattern*species | *F* | 0.007 | 13.975 | 0.225 | 0.816 | 1.939 | 0.228 | 9.677 | 16.292 | 0.681 | 0.189 |
|  | *P* | 0.934 | 0.006 | 0.648 | 0.392 | 0.201 | 0.646 | 0.014 | 0.004 | 0.433 | 0.675 |

SM, Soil moisture; TN, Total nitrogen; NO_3_^-^-N, Nitrate nitrogen; NH_4_^+^-N, Ammonium nitrogen; DON, Dissolved organic nitrogen; OM, soil organic matter; AP, Available phosphorus; Soil C/N, Soil organic carbon/total nitrogen; Litter C/N, Litter carbon/total nitrogen.

**Supplementary Table S2** Results of two-way ANOVAs for the effects of pattern and tree species on EMF alpha diversity of Larch monoculture, Spruce monoculture, Ash-Larch mixture and Ash-Spruce mixture stands at Maoershan Experiment Station, Northeast China.

| Factor | Variable | Chao1 | Sobs | Pielou | Shannon | Simpson |
| --- | --- | --- | --- | --- | --- | --- |
| pattern | *F* | 4.274 | 18.457 | 16.570 | 80.460 | 29.265 |
|  | *P* | 0.073 | 0.003 | 0.004 | < 0.001 | < 0.001 |
| species | *F* | 0.981 | 3.201 | 0.170 | 3.662 | 0.373 |
|  | *P* | 0.351 | 0.111 | 0.691 | 0.092 | 0.275 |
| pattern*species | *F* | 0.176 | 0.934 | 0.732 | 0.624 | 1.767 |
|  | *P* | 0.686 | 0.362 | 0.417 | 0.452 | 0.220 |

Sobs, Number of OTUs observed.

**Supplementary Table S3** Significances and explained variances of environmental covariates (including soil physicochemical properties and litter quality) modeled by EnvFit on the Bray-Curtis dissimilarity of mycorrhizal fungal communities.

| Variable | Arbuscular mycorrhizal fungi | | | | Ectomycorrhizal fungi | | | |
| --- | --- | --- | --- | --- | --- | --- | --- | --- |
|  | NMDS1 | NMDS2 | r^2^ | Pr(>r) | NMDS1 | NMDS2 | r^2^ | Pr(>r) |
| NO_3_^-^-N | -0.07579 | -0.99712 | 0.1644 | 0.584 | -0.87292 | -0.48787 | 0.8067 | 0.001 |
| NH_4_^+^-N | -0.14732 | -0.98909 | 0.4437 | 0.172 | -0.95393 | -0.30003 | 0.2476 | 0.254 |
| DON | -0.06356 | -0.99798 | 0.3371 | 0.288 | -0.93717 | -0.34887 | 0.7213 | 0.004 |
| OM | -0.54372 | 0.83927 | 0.0839 | 0.739 | 0.98766 | 0.15659 | 0.8184 | 0.002 |
| AP | -0.39741 | -0.91764 | 0.0193 | 0.940 | 0.99969 | -0.02502 | 0.0661 | 0.736 |
| TN | -0.96488 | -0.26271 | 0.2444 | 0.428 | -0.24264 | -0.97012 | 0.3163 | 0.192 |
| SCN | 0.23377 | 0.97229 | 0.0659 | 0.809 | 0.88326 | 0.46888 | 0.8614 | 0.001 |
| LCN | -0.45028 | 0.89289 | 0.1283 | 0.642 | 0.79407 | 0.60783 | 0.7657 | 0.003 |
| SM | -0.19987 | -0.97982 | 0.0848 | 0.745 | 0.14914 | -0.98882 | 0.5205 | 0.038 |
| pH | 0.34465 | -0.93873 | 0.4669 | 0.166 | -0.66018 | -0.75111 | 0.5675 | 0.023 |

NO_3_^-^-N, Nitrate nitrogen; NH_4_^+^-N, Ammonium nitrogen; DON, Dissolved organic nitrogen; OM, soil organic matter; AP, Available phosphorus; TN, Total nitrogen; SCN, Soil organic carbon/total nitrogen; LCN, Litter carbon/total nitrogen; SM, Soil moisture.
